# Brazilian cross-cultural adaptation of the DocCom online module: communication for teamwork^1^

**DOI:** 10.1590/1518-8345.1554.2924

**Published:** 2017-09-18

**Authors:** Tatiane Angélica Phelipini Borges, Marli Terezinha Oliveira Vannuchi, Suely Grosseman, Alberto Durán González

**Affiliations:** 2 Doctoral student, Universidade Estadual de Londrina, Londrina, PR, Brazil.; 3 PhD, Adjunct Professor, Departamento de Enfermagem, Universidade Estadual de Londrina, Londrina, PR, Brazil.; 4 PhD, Associate Professor, Departamento de Pediatria, Universidade Federal de Santa Catarina, Florianópolis, SC, Brazil.; 5 PhD, Adjunct Professor, Departamento de Saúde Coletiva, Universidade Estadual de Londrina, Londrina, PR, Brazil.

**Keywords:** Nursing, Communication, Patient Care Team, Computer-Assisted Instruction, Professional Competence

## Abstract

**Objective:**

to carry out the cross-cultural adaptation of DocCom online module 38, which deals with teamwork communication into Portuguese for the Brazilian contexto.

**Method:**

the transcultural translation and adaptation were accomplished through initial translations, synthesis of the translations, evaluation and synthesis by a committee of experts, analysis by translators and back translation, pre-test with nurses and undergraduate students in Nursing, and analysis of the translators to obtain the final material.

**Results:**

in evaluation and synthesis of the translated version with the original version by the expert committee, the items obtained higher than 80% agreement. Few modifications were suggested according to the analysis by pretest participants. The final version was adequate to the proposed context and its purpose.

**Conclusion:**

it is believed that by making this new teaching-learning strategy of communication skills and competencies for teamwork available, it can be used systematically in undergraduate and postgraduate courses in the health area in Brazil in order to contribute to training professionals, and also towards making advances in this field.

## Introduction

Teamwork is based on the interaction of a group of people who perform activities and actions to achieve common goals. However, in the health area, where the care provided to human beings is the product of their actions, the work process must mainly be permeated by communication and interpersonal relationships^1^ ^-^ ^2^.

In this way, health team members, and specifically of nursing teams, need to articulate technical and scientific knowledge in order to share the planning and execution of actions with mutual collaboration, interaction, professionalism and co-responsibility.

In the team work performed by nursing, communication is used beyond the action of just collecting and transmitting information; it is used as a tool and a strategy for therapeutic management. The interaction between nurses and the team, as well as between the team and the patient and their family members, involves propagating attitudes and actions with an intentionality full of sensitivity and empathy; which are fundamental concepts in the act of caring^3^. 

From this perspective, it is expected that nurses who are responsible for teams have the skills and competencies to perform quality care management. Among them, communication, decision-making, negotiation, teamwork, interpersonal relationships and creativity stand out. Therefore, it is necessary to incorporate curricula for training and improving communication skills in undergraduate and postgraduate programs to be used as care strategies^4^ ^-^ ^5^.

Investments in new information and communication technologies have been widely discussed; however, these pedagogical tools allied to teaching are still little employed in Brazil. The use of new technologies would facilitate these advances, supporting teaching-learning processes and studies to evaluate results^6^ ^-^ ^7^. 

In this context, one of the models for teaching communication skills is the online platform *DocCom*, which was developed by a team of American professors linked to the American Academy of Communication in Healthcare (AACH) and the Drexel University College of Medicine (DREXELMED), Philadelphia, USA, available at http://webcampus.drexelmed.edu/doccom. The platform contains multimedia resources that include evidence-based theory and video demonstrations, which should be used in conjunction with group discussions and hands-on activities such as inpatient interviews and role plays. This technique makes it possible to place the individual in face of situations similar to real ones^8^ ^-^ ^9^.

*DocCom* is used by American, Japanese and Australian universities by medicine students and residents for teaching communication skills related to the doctor/patient relationship, biopsychosocial aspects of care and teamwork^10^. Although online *DocCom* modules have been developed and applied in the medical field, some modules deal with topics that are transversal to courses in the health area.

In Brazil, teaching communication skills is offered in some universities in the health area, mainly in medical courses, however, in general it occurs by individual initiatives. The structuring of a national proposal to be used in training health professionals is desirable, considering the need to develop communication skills and teamwork for these professionals^11^ ^-^ ^12^. 

Thus, this study aimed to perform the cross-cultural adaptation of *DocCom* online module 38 on teamwork communication for Portuguese for the Brazilian contexto.

## Method

This is a methodological study regarding the translation and cross-cultural adaptation of *DocCom* online module 38^13^ ^)^ from the English language to Portuguese for the Brazilian contexto. Authorization for the translation and cross-cultural adaptation of *DocCom* online module 38: communication for teamwork, was granted prior to development of this study by the associate coordinator and director of teaching and Clinical Skills Assessment of the Medical Education Division of the DREXELMED, who owns the copyrights to *DocCom* ^10^ ^,^ ^14^.

The *DocCom* platform contains 42 online modules. Module 38 contains texts on leadership and teamwork, communication skills and meeting processes, 16 skills’ demonstration videos lasting approximately three minutes each, a questionnaire with cognitive questions to evaluate the acquired knowledge and another to assess the achievement of the learning objectives at the end of the module^14^. 

We emphasize that *DocCom* online module is not a psychometric instrument, meaning that it is not a measuring instrument of a phenomenon, which in this case would need to be adequate for different cultures; it is a module for teaching-learning skills. In this sense, considering that no methodologies have been found in national and international literature contemplating the translation and adaptation of computerized materials simultaneously involving a textual part and videos without losing the essence of the concepts from the original language, we have chosen to follow the steps proposed internationally by the authors^15^ ^-^ ^17^ for translating instruments, which comprise: 1) translation of the original language into the target language by two professionals; 2) synthesis of the translations; 3) evaluation of the translation synthesized by a group of judges; 4) back-translation and 5) pre-test. 

**Figure 1:**
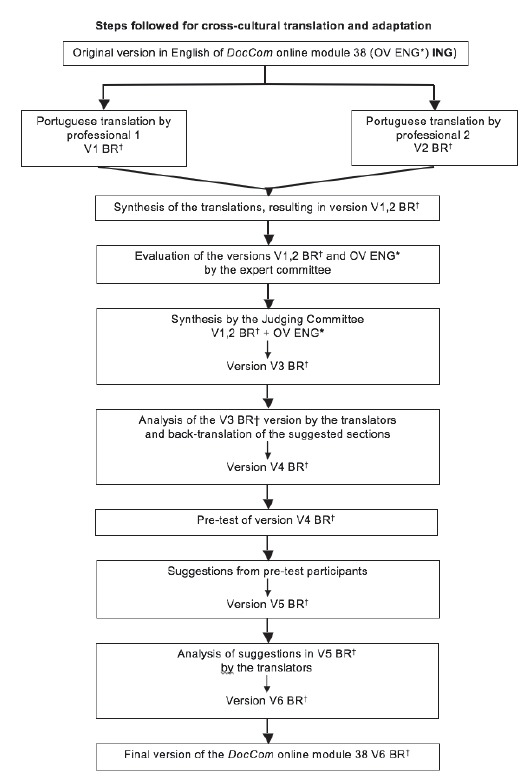
Cross-cultural translation and adaptation process of DocCom online module 38 into Portuguese for the Brazilian contexto. Londrina, PR, Brazil, 2015

Figure 1 shows the steps followed for cross-cultural translation and adaptation of *DocCom* online module 38.

*OV ENG= Original Version in English/†V nº BR= Version with its respective number in Portuguese for the Brazilian contexto

The authors’ study^15^ point out that the steps mentioned above should be understood as guidelines for better quality of the translation and cross-cultural adaptation process. Based on this, we justify not performing the back-translation step for all material, but only the suggested sections for changes after evaluation by the judging committee and the pre-test.

The translation was carried out by 2 translators fluent in English, where only one of them was aware of the objectives of the study. The Portuguese for the Brazilian contexto translation of the *DocCom* online module 38 resulted in the Brazilian version 1 (V1 BR) and Brazilian version 2 (V2 BR). Both were compared and discussed by the translators, resulting in the Brazilian version 1,2 (V1,2 BR).

An evaluation and comparison of the translated and synthesized version (V1,2 BR) with the original English version (OV ENG) was carried out by a committee of judges. They were guided by an instrument that contained a presentation of the study, its objectives and all instructions on how they should proceed for evaluating the material, in addition to contact information from the researchers in case of any doubts.

The judging committee was composed of 11 specialists in the area of Nursing Management, of which 2 are professors with Doctorate Degrees, 3 are professors with Masters Degrees, and 6 are former residents of the Nursing Services Management. All the participants were nurses and had knowledge about the subject and the English language. The main function of the judging committee was to compare the original material with the translated version, evaluating the semantic, idiomatic, cultural and conceptual equivalences^15^ ^-^ ^17^.

Semantic equivalence evaluates the meaning of words in an attempt to preserve their original meaning; idiomatic equivalence analyzes formulation of expressions and colloquialism equivalent in the target language; cultural equivalence refers to everyday terms and situations that differ between cultures; and conceptual equivalence refers to words that have cultural meanings^15^ ^-^ ^17^. These parameters were essential so that the adequacies, receptivity and the judges’ opinions involved in this phase of the study could be verified, along with the purpose of evaluating the pertinence of the module for teaching-learning of students in the health area.

After their individual analysis, the judges committee met with the researchers to discuss the items and their proposed amendments. The meeting lasted two and a half hours and was recorded in audio so that it could subsequently aid in typing all the suggestions for changes.

After consensus of the judging committee regarding the items, a compilation with all the recommendations regarding textual content and videos was made resulting in the Brazilian version 3 (V3 BR). This version was sent for appreciation to the translators who carried out the back-translation of the suggested sections, resulting in Brazilian version 4 (V4 BR). 

The next step was the pre-test, which consisted of using the material with a group of subjects in order to ensure that the adapted version was equivalent to that of the original version. In addition, it assisted in detecting errors and ambiguities, and helped to ratify understanding the content^15^ ^-^ ^17^.

The pre-test version (V4 BR) was carried out with a group of nurses and undergraduate Nursing students. Subsequent to the pre-test, all the suggestions regarding modifications of words and/or phrases of the items considered as necessary changes were collected, thus resulting in the Brazilian version 5 (V5 BR). This version was then sent to the translators for analysis and back-translation of excerpts indicated in the pre-test, resulting in the Brazilian version 6 (V6 BR), understood as the final version of the *DocCom* online module 38, translated and adapted to the Portuguese for the Brazilian contexto language.

It should be noted that all ethical and legal principles of Resolution 466/12 of the National Health Council^18^ were met and the research was approved by the Research Ethics Committee Involving Human Beings, under Opinion 181/2014 and *CAAE* 34827314.8.0000.5231 *CEP/UEL*. All study participants signed the Free and Informed Consent Form (*TCLE*).

## Results

The first translation step (V1 BR and V2 BR) and synthesis (V1,2 BR) were performed without significant changes.

Versions (V1,2 BR) and (OV ENG) were sent to the judging committee for them to compare the versions regarding semantic equivalence. Complete access to module 38 materials of the *DocCom* online platform were made available to the judges for them to be able to view and analyze the videos in the original and the translated versions, so that they could quash any doubts regarding the content as necessary. 

The percentage of items suggested for changes on *DocCom* online module 38 was first calculated based on individual judges’ notes. A group discussion was subsequently held to compare the suggested changes in order to reach a consensus regarding rephrasing.

In total, the judges suggested 56 words and/or phrases (V3 BR) to undergo changes to achieve textual equivalence; of these, 5 were semantic changes, 28 idiomatic changes, 5 cultural changes, 18 conceptual changes and 15 grammatical concordances. The changes suggested by the judges were considered to be few in view of the extent of the evaluated content.

The highest indexes for suggestions among the judges were, respectively, semantic ones: 45% (item 2), 54% (item 1) and 73% (items 4 and 5); idiomatic ones: 45% (item 22), 54% (items 27 and 33), 64% (items 9, 16, 18 and 28), 73% (item 20), 82% (Items 6 and 7) and 91% (Items 8 and 11); cultural ones: 91% (item 35) and 100% (items 36, 37 and 38); and conceptual ones: 45% (items 41 and 45), 54% (item 47), 64% (item 44), 73% (items 42, 43, 46 and 49) and 82% (items 39, 40, 55, 56 ). The remaining items had percentages equal to or below 44% for all equivalences.

After discussing the 56 items suggested above, a percentage of agreement was reached between the judges for modifications of all items, with the majority being equal to or greater than 82%, and some reaching 100% agreement according to the justification that altering them would make the context clearer and easier to understand. 

Next, a compilation of the suggestions was organized and sent to the translators. Of the 56 words and/or sentences, 13 were not accepted according to the justification that the essence of the original concepts of the module should be maintained after back-translation of the suggestions; of these, 8 were idiomatic, 1 cultural and 4 conceptual. The remaining suggestions were accepted and inserted in the new version (V4 BR) of the *DocCom* online module 38 for later realization of the pre-test. 

The pre-test was carried out after the end of the verification stage for the version (V4 BR) by the judges, in which it was considered relevant. The pre-test was carried out with 9 individuals, with 3 nurses and 6 scientific initiation students in their initial 3 semesters of the undergraduate Nursing course. 

Thus, a copy of the textual content (V4 BR) was given to each participant of this phase, along with an instrument guiding them on how the material should be read and of its main purpose, while also explaining how the annotations regarding the items and unclear contents should be done. 

Pretest participants suggested 8 grammatical corrections, 13 phrase and/or word changes regarding the equivalences; 3 were semantic, 8 idiomatic and 2 cultural. All were transcribed after being analyzed by the researchers, resulting in version (V5 BR).

Version (V5 BR) was then sent to the translators for consideration as to the relevance of the suggestions. Those of grammatical order were accepted because they were verbal and/or nominal concordances, attributing greater clarity to the sentences. Regarding the 13 equivalences, 3 were not accepted by the translators (of which 2 were semantic and 1 was idiomatic), by their justification that the meaning of the original concepts of the module should be maintained and ratified after the back-translation of the suggestions. The other recommendations were followed, resulting in the final version (V6 BR) of module 38.

The judges’ and pretest participants’ considerations in relation to videos were minimal. Only the caption of one video was behind in relation to the actors’ speech. The changes regarding replacing sentences and/or words were few as mentioned in the suggestions of the textual part. 

## Discussion

Technology insertion in the most diverse spaces, services and organizations is undeniable. Thus, it is increasingly difficult to overlook the connection of Information and Communication Technology (ICT) in teaching and learning environments, since the student arrives at the school bringing with them an array of technological devices, and in addition to that, education has undergone numerous transformations due to the current demands in the labor market by professionals better prepared to face these changes^19^. 

The increase of computerization in teaching environments should prioritize the appropriation and use of new tools in order to build individual knowledge. The new teaching and learning scenarios and the enhancement of knowledge through ICT becomes feasible by increasing educational practices allied to evidence-based theory, and videos with dramatizations of theoretical-practical situations followed by face-to-face and online discussions in small groups guided by a tutor. These strategies allow the student to act autonomously in the search for transformations in their praxis; and not just as a receiver, repeating tasks into a tool^20^.

Investing in the teaching-learning processes is necessary for advances to be made in improving the care process, in learning to learn, to be and to live together. Thus, electing new ICTs through computerization can facilitate such advances based on the teaching-learning processes, as well as on studies that evaluate its applicability and effectiveness^21^.

Therefore, the methodological steps of translation and transcultural adaptation of the module were carried out in a satisfactory way. The translation and back-translation of the excerpts, where alterations were recommended, allowed for identifying misunderstandings and discrepant interpretations of words whose concepts and meanings were divergent from the originals. 

The discussion among the judging committee made it possible to analyze, in consensus, the semantic, idiomatic, cultural and conceptual equivalence of 56 suggestions for replacing/changing words and/or phrases.

We emphasize the importance of including the judging committee to evaluate the content of the *DocCom* online module 38, especially due to the complexity in adapting the terms and concepts employed by Americans in relation to Brazil. It is necessary to respect the singularities of teaching and the pedagogical context to ensure that its use can provide the expected benefits^6^ ^,^ ^14^.

Thus, carrying out the individual evaluation of the material was pertinent, as well as analyzing the suggestions for modifiable items only after, in order to appreciate the recommendations in group discussion of the content that caused doubts, so that a consensus could be reached, resulting in the back-translation version and then the pre-test. 

Most of the participants’ suggestions in the pre-test were related to idiomatic and semantic equivalences. The implemented changes contributed to a better understanding of the words used, thereby facilitating understanding of the phrases and concepts, such as the expression “team experience”, which was replaced by “teamwork experience”, considering the actions developed with the patient in teamwork.

Teamwork in nursing is constituted by the set of knowledge, experiences and interpersonal relationships, since its work is based on human relations of the team, of the care to the patient and of sharing interdisciplinary work^3^ ^,^ ^20^ ^-^ ^21^.

After the last changes carried out according to the suggestions from pretest participants, the researchers along with the translators came to a consensus that the *DocCom* online module 38 would not require further analysis or changes (V6 BR), thus being suitable to be used and implemented in the teaching-learning process of communication skills for teamwork in different health areas.

The fact that the pre-test was submitted to a small group of people only from the nursing area can be seen as limiting factor of this study. However, the members of the group in question had different levels of knowledge in the nursing area; thus, we understand that the main purpose of this stage was to analyze the material regarding its content clarity. This may give rise to a new content evaluation in other health areas, so that it can be verified that the employed concepts are also understandable by other professionals.

There is no doubt that exploring new methodological alternatives and innovative practices to promote teaching and learning lead to considerable and positive transformations for education, especially in higher education. Aligning teaching with technology helps to arouse the interest of students in new methods while in the pursuit of their own learning, as well as a better understanding, thereby aligning theory and practice^22^ ^-^ ^23^. 

The Brazilian version (V6 BR) of the *DocCom* online module 38 contemplated the originally addressed content, and was adapted to the proposed context.

## Conclusion

The cross-cultural translation and adaptation process of the *DocCom* online module 38 - communication for teamwork - for the Brazilian culture followed the internationally recommended methodological guidelines and was successfully completed, resulting in adequate material in Portuguese for the Brazilian contexto, comprehensible and with content concordance, which can be applied to other courses in the health area, in addition to medicine. 

After completion of the previous procedures, the next step is using the *DocCom* online module 38 in teaching-learning communication skills in undergraduate and residential courses in health, especially medicine and nursing, so that it is possible to verify the validity and understanding of the covered content in practice.

Considering that the teaching of skills and competencies in Brazilian universities still does not occur systematically, unlike in other countries where efforts are being applied to stimulate this modality of education, this study contributes to making this new strategy available for teaching-learning skills and communication skills for teamwork, so that it can be used systematically in undergraduate and postgraduate courses in the health area.

Although the use of computerized tools in isolation may not substantially contribute to improving students’ knowledge, its use combined with theory, videos with dramatizations and subsequent group discussion and role-play can cooperate in a meaningful way for the meaningful learning of the students, which is the case of *DocCom* online module 38. 

Despite the fact that *DocCom* online module 38 was originally designed for the medical field, it was translated, adapted and applied to nursing in this study because the subjects covered in the module are pertinent to the work in several undergraduate and postgraduate courses in the health area, and because they meet the skills and competencies advocated by the National Curricular Guidelines of these courses.
